# *CDC73* mutational status and loss of parafibromin in the outcome of parathyroid cancer

**DOI:** 10.1530/EC-13-0046

**Published:** 2013-11-18

**Authors:** Filomena Cetani, Chiara Banti, Elena Pardi, Simona Borsari, Paolo Viacava, Paolo Miccoli, Liborio Torregrossa, Fulvio Basolo, Maria Rosa Pelizzo, Massimo Rugge, Gianmaria Pennelli, Guido Gasparri, Mauro Papotti, Marco Volante, Edda Vignali, Federica Saponaro, Claudio Marcocci

**Affiliations:** 1 Department of Clinical and Experimental MedicineUniversity of PisaVia Paradisa 2, PisaItaly; 2 Section of Pathology, Department of OncologyUniversity of PisaVia Paradisa 2, PisaItaly; 3 Department of Surgical Medical and Molecular Pathology and Critical AreaUniversity of PisaPisaItaly; 4 Surgery Unit, Surgical Oncology and Gastroenterology SciencesUniversity of PaduaPaduaItaly; 5 MedicineUniversity of PaduaPaduaItaly; 6 General Surgery 3 and Esophageal SurgeryUniversity of TurinTurinItaly; 7 Clinical and Biological SciencesUniversity of TurinTurinItaly

**Keywords:** primary hyperparathyroidism, parathyroid tumorigenesis, immunostaining, survival

## Abstract

Inactivating mutations of the *CDC73* tumor suppressor gene have been reported in parathyroid carcinomas (PC), in association with the loss of nuclear expression of the encoded protein, parafibromin. The aim of this study was to further investigate the role of the *CDC73* gene in PC and evaluate whether gene carrier status and/or the loss of parafibromin staining might have an effect on the outcome of the disease. We performed genetic and immunohistochemical studies in parathyroid tumor samples from 35 patients with sporadic PC. Nonsense or frameshift *CDC73* mutations were detected in 13 samples suitable for DNA sequencing. Six of these mutations were germline. Loss of parafibromin expression was found in 17 samples. The presence of the *CDC73* mutation as well as the loss of parafibromin predicted a high likelihood of subsequent recurrence and/or metastasis (92.3%, *P*=0.049 and 94.1%, *P*=0.0017 respectively), but only the latter was associated with a decreased overall 5- and 10-year survival rates (59%, *P*=0.107, and 23%, *P*=0.0026 respectively). The presence of both the *CDC73* mutation and loss of parafibromin staining compared with their absence predicted a lower overall survival at 10- (18 vs 84%, *P*=0.016) but not at 5-year follow-up. In conclusion, loss of parafibromin staining, better than *CDC73* mutation, predicts the clinical outcome and mortality rate. The added value of *CDC73* mutational analysis is the possibility of identifying germline mutations, which will prompt the screening of other family members.

## Introduction

Primary hyperparathyroidism (PHPT) is one of the most common endocrine diseases (1). It is usually a sporadic disorder, but in a minority of cases (<10%) it is a part of hereditary syndromes, namely multiple endocrine neoplasia type 1 and 2A, hyperparathyroidism–jaw tumor syndrome (HPT–JT), and familial isolated hyperparathyroidism (2). Sporadic PHPT is due to a single parathyroid adenoma in 80–85% of cases, multiglandular hyperplasia in 10–15%, and carcinoma in <1%.

The histological diagnosis of parathyroid carcinoma (PC) is currently restricted to lesions showing unequivocal extra-parathyroidal growth, as evidenced by perineural invasion, full thickness capsular invasion with growth into adjacent tissues, extratumoral vascular invasion, or metastasis (3). A subset of parathyroid tumors (atypical adenomas) shows pathological features of PC such as trabecular growth, fibrous bands, marked cellular atypia, and increased mitotic activity, in the absence of invasive growth. Thus, the distinction between benign and malignant parathyroid tumors cannot be definitively established by histology, unless there is evidence of invasion of extratumoral vessels, perineural spaces, or surrounding tissues (thyroid and other adjacent structures) (4). However, it is noteworthy that there are patients who develop distant metastases during the course of the disease who did not show either extratumoral vascular (40%) or capsular (10–15%) invasion during histological examination of the original parathyroid tumor (5, 6).

Advances in the knowledge of the molecular pathogenesis of PC have been made as a result of the cloning of *CDC73*, previously known as *HPRT2* the gene responsible for HPT–JT syndrome, in which there is a high prevalence of PC (7). Somatic inactivating mutations of the *CDC73* gene have also been reported in up to 70% of apparently sporadic metastatic PC (8, 9, 10, 11, 12, 13, 14, 15, 16, 17). Notably, in about one-third of patients, the mutations were germline (8, 12, 14, 15). A lower prevalence (15%) of *CDC73* mutations in PC classified as malignant only on the basis of histological criteria (namely the presence of angioinvasion, with or without capsular invasion and/or distant metastases) has been reported by Haven *et al*. (18).

Following the demonstration of *CDC73* mutations in PC, several studies were carried out to evaluate whether immunostaining of parafibromin, the gene product, might have some diagnostic utility. Diffuse or focal loss of parafibromin expression as determined by immunohistochemistry was found in the majority of PC, in one-third of atypical adenomas, and very rarely in parathyroid adenomas (8, 9, 10, 11, 12, 13, 19, 20, 21, 22, 23, 24).

The aim of this study was to further investigate the role of the *CDC73* gene in PC and evaluate whether the gene carrier status and/or the loss of parafibromin staining might have an effect on the outcome of the disease.

## Subjects and methods

### Patients

We studied 35 patients with apparently sporadic PC, collected between 1987 and 2011. Patients underwent parathyroidectomy (PTx) at the Departments of Surgery of the University of Pisa (*n*=10), Padua (*n*=8), Turin (*n*=15), and Genoa (*n*=2). Clinical and biochemical data of interest were obtained from medical records examined at the end of 2012. The study was approved by our Internal Review Board and informed consent was obtained where required.

### Tissue samples

Thirty-five tumor specimens (26 paraffin-embedded samples and nine fresh-frozen tissues) were studied. All samples met the histological diagnosis of PC according to the recent World Health Organization classification (3).

### *CDC73* gene analysis

Genomic DNA was isolated by standard methods from fresh or paraffin-embedded parathyroid tissues and peripheral blood leucocytes or control tissue of patients in whom the mutation was detected. The entire coding region and splice site junctions of the *CDC73* gene were PCR amplified and directly sequenced as previously described (14).

### Immunohistochemistry

Immunohistochemistry was performed as previously described (12). In brief, archival sections were deparaffinized in xylene and rehydrated in alcohol. The sections were incubated for 1 h with the primary MAB (clone sc-33638 from Santa Cruz Biotecnology), used at the dilution of 1:50. The antibody is directed against the portion of the protein corresponding to amino acid positions 87–100. The sections were then incubated with biotin-labeled secondary antibody (dilution 1:500) and subsequently with avidin–biotin complex (Vector Burlingame, Burlingame, CA, USA) for 30 min each. Sites of binding were visualized using 3,3-diaminobenzidine as the chromogen. Finally, sections were counterstained with hematoxylin, dehydrated, and mounted. Five normal parathyroid specimens obtained from normocalcemic patients who had undergone surgery for nodular goiter were used as controls. In each experiment adjacent stromal/endothelial cells served as an internal positive control. Parafibromin negative controls consisted of experiments in which the primary antibody was omitted. For each tumor sample, six different sections were analyzed. Cells were scored as positive if specific nuclear staining was detected, independently of the intensity of staining. Tumor staining was quantified according to the percentage of cells showing specific nuclear staining. Each section was evaluated by two independent observers (P Viacava and L Torregrossa) who were blinded to the initial pathological diagnosis and clinical outcome. When the assessment of the percentage of positive cells differed between the two observers, the disagreements were resolved by reaching a consensus after joint review using a conference microscope.

### Statistical analysis

Results were expressed as means (±s.d.) (for normally distributed continuous variables), median and interquartile range (for non-normally distributed continuous variables), or prevalence, as appropriate. Differences among patient groups were tested by Mann–Whitney *U* test, *χ*^2^ test, or Fisher test, as appropriate. Differences in the overall survival (time to death of the disease) among patient groups were evaluated by the Kaplan–Meier method, and *P* values were calculated by the log-rank test. A *P* value of <0.05 was considered as statistically significant.

## Results

### Patients

The clinical and biochemical characteristics of patients are summarized in Tables 1 and 2. The mean age at diagnosis was 45 years and there was no gender preference. The majority of patients had kidney and bone involvement. Twenty-five (71.4%) patients had recurrence and/or metastases and 18 of them died of the disease after a median follow-up of 5.5 years (interquartile range 4, 8). Ten patients had no evidence of recurrence and/or metastases and were all alive after a median follow-up of 9.5 years (7, 13).

### *CDC73* genetic analysis

**Figure 1:**
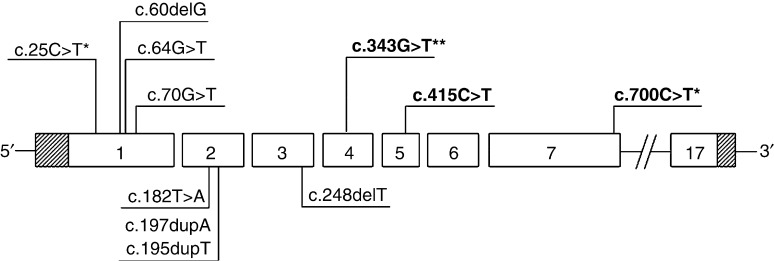
A schematic representation of the *CDC73* gene showing the position of the different identified mutations. Mutations are designated according to the latest nomenclature recommendations of the Human Genome Variation Society. Mutations in bold are germline. Mutations found in two or three patients are indicated by (*) and (**) respectively.

The genetic analysis of the entire coding sequence and splice sites was performed in 32 out of 35 (91%) tumor samples. In the remaining three samples, despite using different protocols for DNA extraction, DNA sequencing was incomplete and therefore these samples were excluded from subsequent analyses (Table 2). A *CDC73* mutation was detected in 13 out of 32 (41%) tumors; a double mutation was found in two cases (numbers 27 and 43). Sample no. 43 harbored two unreported frameshift mutations, 1-bp deletions in exons 1 (c.60delG) and 3 (c.248delT), which predict an alteration of the reading frame with a truncation at codons 20 (Val20ValfsX6) and 83 (Ile83IlefsX26). All mutations resulted in a premature stop codon. Five mutations were localized in exon 1, three in exon 4, three in exon 2, two in exon 7, and one in exon 5 (Fig. 1).

Sequencing of peripheral blood leucocytes or control tissue from patients carrying the *CDC73* mutation showed that six mutations (E115X in three cases, R234X in two, and R139X in one) were germline. Patients carrying the same mutation were apparently unrelated, even though a common ancestor could not be excluded. There was no statistically significant difference in the age at diagnosis between patients carrying a somatic or a germline mutation, even though the mean age was higher in the former group (50±8 vs 38±16, *P*=0.094).

The sensitivity, specificity, positive and negative predictive values of the presence of *CDC73* mutation are reported in Table 3.

### Immunohistochemistry

**Figure 2:**
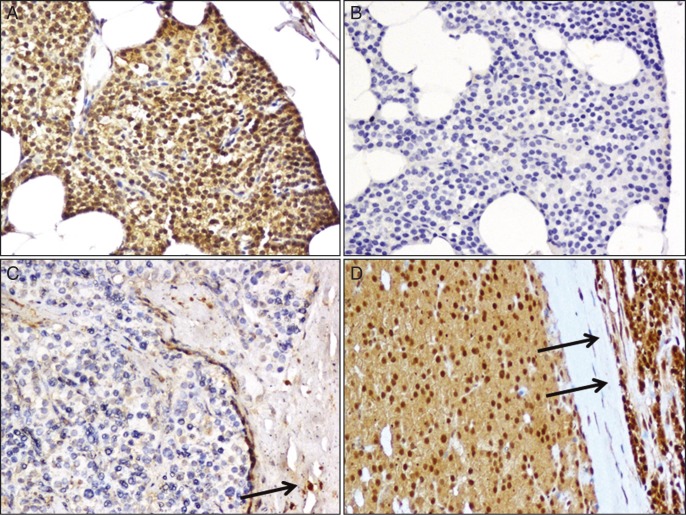
Immunohistochemical staining of parafibromin. (A) Normal parathyroid gland. The parathyroid cells show a diffuse nuclear immunoreactivity associated with a moderate cytoplasmatic staining (×200). (B) Normal parathyroid gland, negative control (omission of primary antibody). No nuclear staining is evident (×200). (C) A representative case of parathyroid carcinoma scored as negative. The neoplastic cells are completely negative for parafibromin. The positive staining of non-neoplastic stromal cells (arrow) provides an internal positive control (×200). (D) A representative case of parathyroid carcinoma scored as positive. The neoplastic cells show a diffuse nuclear and cytoplasmatic immunoreactivity for parafibromin. The adjacent rim of normal parathyroid tissue (arrows) shows a diffuse immunoreactivity for parafibromin (×200).

Nuclear parafibromin staining was evident in almost all cells in the normal parathyroid specimens as well as in the endothelial cells within the parathyroid tumors (Fig. 2).

Immunohistochemistry was performed in 34 out of 35 specimens (Table 2). Immunostaining for parafibromin was negative (percentage of nuclear staining in <5% of cells) in 17 out of 34 (50%) tissue samples. The remaining 17 tumor samples were scored as positive, with a percentage of positive cells ranging between 10 and 80% (median (interquartile range) 30 (10, 30)). A faint cytoplasmic staining was also observed in the normal parathyroid gland as well as in some parathyroid tumors. Representative cases are shown in Fig. 2.

The sensitivity, specificity, positive and negative predictive values of the loss of parafibromin immunostaining are reported in Table 3.

### Impact of the *CDC73* or parafibromin status on the outcome

**Figure 3:**
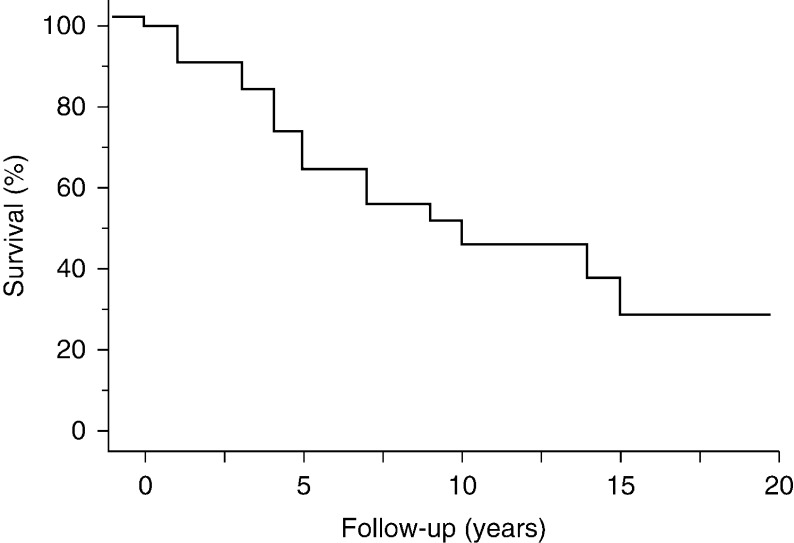
Overall survival in 35 patients with parathyroid carcinoma.

The median duration of follow-up was 7 years (interquartile range 4, 11). The survival at 1, 5, 10, and 15 years was 97, 72, 50, and 36% respectively (Fig. 3).

#### *CDC73* mutation

**Figure 4:**
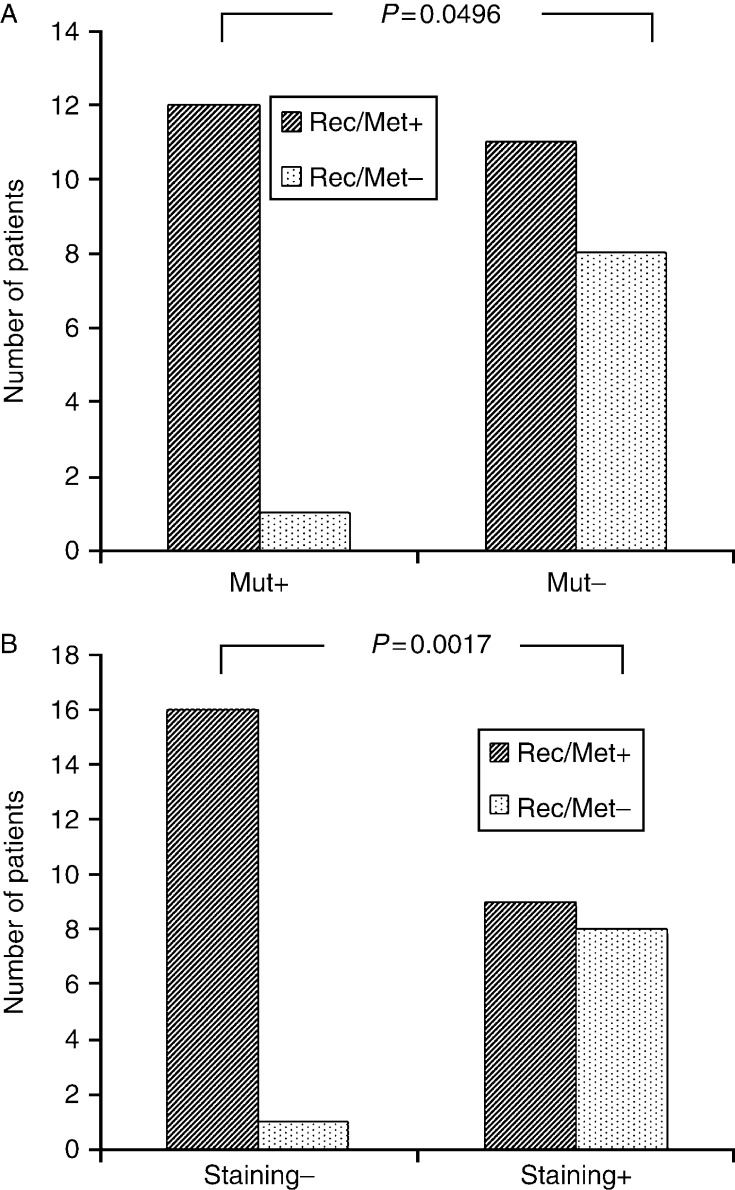
(A) Effect of *CDC73* mutational status on local recurrence and/or metastases in 32 patients with parathyroid carcinoma. Mut+, mutation positive; Mut−, mutation negative; Rec/Met+, development of recurrence and/or metastases; Rec/Met−, no development of recurrence and/or metastases. (B) Correlation of parafibromin staining results with local recurrence and/or metastases in 34 patients with parathyroid carcinoma. Staining−, loss of parafibromin; Staining+, retained parafibromin expression; Rec/Met+, development of recurrence and/or metastases; Rec/Met−, no development of recurrence and/or metastases.

**Figure 5:**
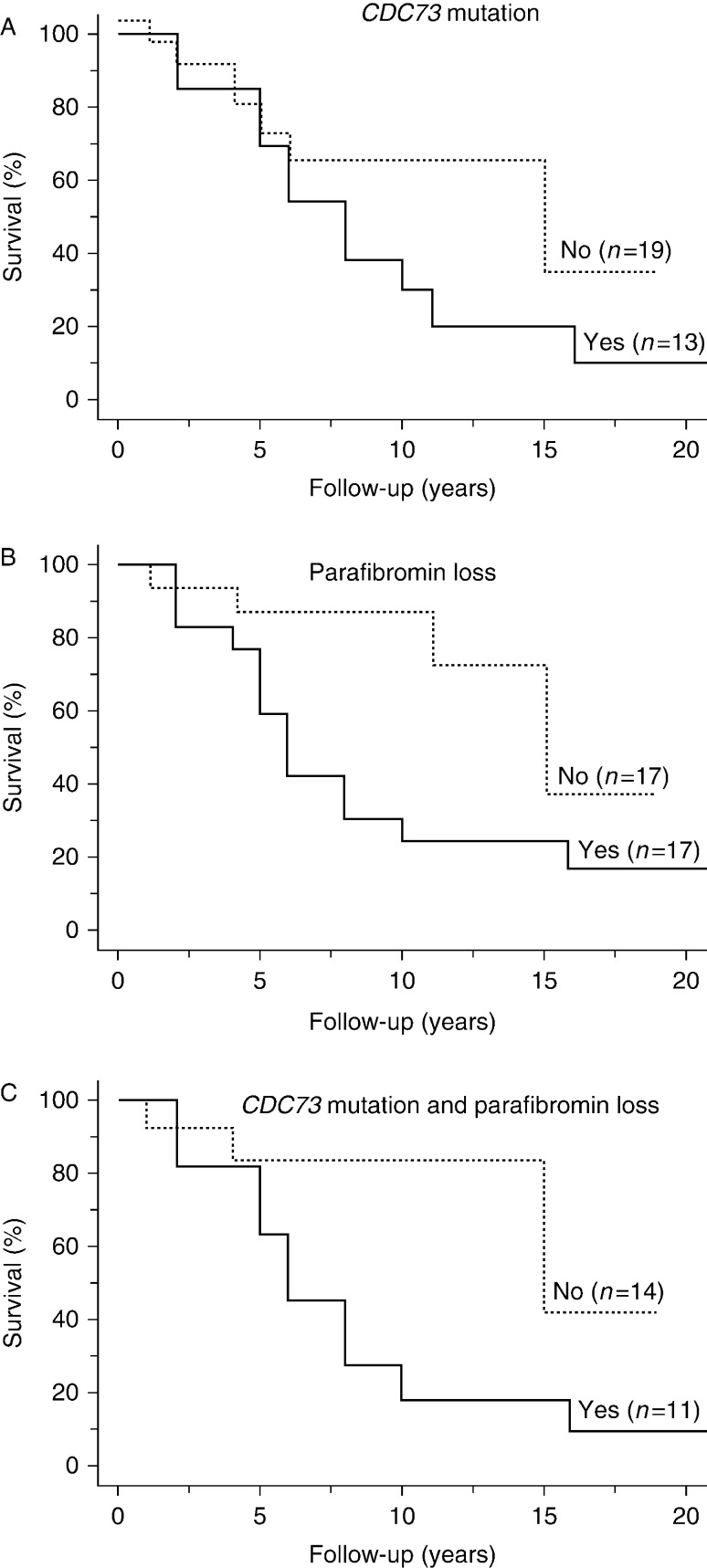
(A) Survival rates according to the presence or absence of *CDC73* mutation. The 10-year survival rates did not differ significantly between the two groups of patients. (B) Survival rates according to the loss of parafibromin. Loss of parafibromin staining was associated with a statistically significant decrease in the 10-year survival. (C) Survival rates according to the presence or absence of *CDC73* mutation and loss of parafibromin. The presence of both abnormalities was associated with a statistically significant decrease in the 10-year survival.

*CDC73* mutational data were available in 32 out of 35 patients. As shown in Fig. 4A, there was a borderline statistically significant association between the mutational status and the outcome. In particular, the presence of the *CDC73* mutation predicted a high likelihood of subsequent recurrence and/or metastasis (92.3%; *P*=0.049). However, the overall 5- and 10-year survivals did not differ between patients carrying or not carrying the *CDC73* mutation (*P*=0.971 and *P*=0.328 respectively; Fig. 5A).

As mentioned earlier, six out of 13 patients carried a *CDC73* germline mutation. We found that the type of mutation (somatic or germline) had no effect on the outcome as all but one patient with germline mutation had recurrence and/or metastases. Moreover, there was no statistically significant difference in the survival rate between patients carrying a somatic (none out of seven) or a germline (two out of six) mutation (*P*=0.192), nor in the mean time elapsed between diagnosis and death (7 years in both groups).

#### Parafibromin status

Parafibromin immunostaining data were available in 34 out of 35 patients. As shown in Fig. 4B, there was a statistically significant association between the immunostaining results and the outcome. In particular, the loss of parafibromin predicted a high likelihood of subsequent recurrence and/or metastasis (94.1%, *P*=0.0017). Moreover, there was an inverse statistically significant association between mortality and the percentage of positive cells (*P*=0.006). The overall 5-year survival did not differ between patients with loss of parafibromin staining compared with patients with retained parafibromin expression (59 vs 87%, *P*=0.107; Fig. 5B). Conversely, the 10-year survival was significantly lower in the former than in the latter group (23 vs 87%, *P*=0.0026).

#### Combined effect of *CDC73* mutation and parafibromin status

**Figure 6:**
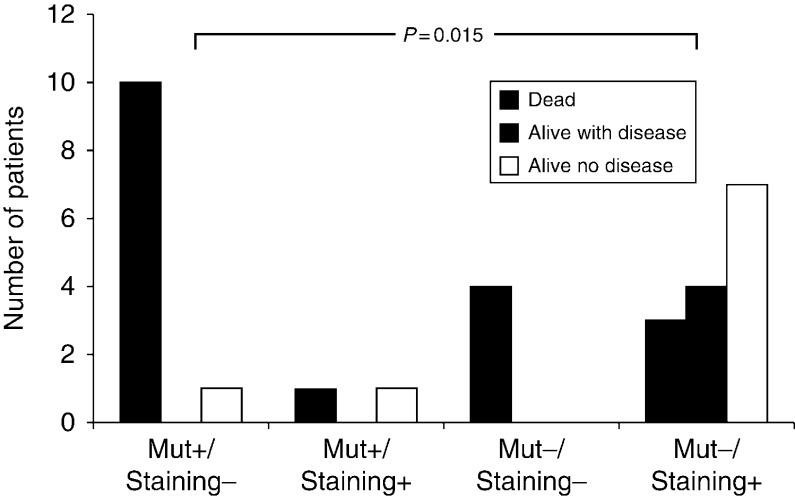
Combined effect of *CDC73* mutational and parafibromin immunostaining results on the outcome in 31 patients with parathyroid carcinoma.

*CDC73* mutational and parafibromin immunostaining data were available for 31 patients. *CDC73* mutation associated with the loss of parafibromin was found in 11 tumor samples and either *CDC73* mutation or loss of parafibromin in six. No *CDC73* mutation or loss of parafibromin staining was observed in the remaining 14 tumor samples. As shown in Fig. 6, there was a statistically significant association between *CDC73* mutation/parafibromin status and the outcome of PC (*P*=0.015). Indeed, ten out of the 11 patients with mutated tumors and loss of parafibromin died of the disease. On the other hand, the majority of patients who had neither *CDC73* mutation nor loss of parafibromin staining were still alive and free of disease (seven out of nine) after a median follow-up of 10 years (interquartile range 7, 19), or still alive but with the disease (*n*=4) after a median follow-up of 4.5 years (interquartile range 3, 6.5).

The overall 5-year survival in the 11 patients carrying the *CDC73* mutation and showing loss of parafibromin staining did not differ from that of the 14 patients with any of these negative prognostic factors (64 vs 84%, *P*=0.337) (Fig. 5C). Conversely, the 10-year survival was significantly lower in the former than in the latter group (18 vs 84%, *P*=0.016).

## Discussion

This study was undertaken to shed light on the molecular mechanisms involved in parathyroid cancer development and metastatic spread. Current evidence indicates that the *CDC73*, the gene responsible for HPT–JT syndrome, which is characterized by a high prevalence of PC, might be a candidate gene. Abnormalities of the *CDC73* gene and its protein, parafibromin, were reported in several series of PC, but their rate differed according to the diagnostic criteria used in different studies (4, 5, 6).

Mutations of the *CDC73* gene were detected in up to 75% of PC from patients who had local invasion and/or metastases at initial diagnosis or during the follow-up. Conversely, a lower rate (15%) was found in a series which included patients who fulfilled the histological diagnosis of PC, but had incomplete follow-up data (18). Based on these findings, it might be hypothesized that patients whose tumors carry the *CDC73* mutation, as compared with those who do not, might have a worse prognosis.

Herein, we confirm that *CDC73* mutations are rather common (48%) in patients with PC. The mutations were scattered along the entire coding region of the gene, but 60% of them were located in exons 1, 2, and 7, the sites harboring up to 85% of mutations reported so far (25). As described in other series (8, 12, 14, 15), 40% of mutations were germline. The percentage of *CDC73* mutation-positive tumors was lower than we previously detected in patients with PC (82%) who had local invasion and/or metastases at initial surgery or during the follow-up (12, 14). In this study, this apparent discrepancy is probably due to the inclusion of patients (15 out of 35) whose diagnosis of PC was only based on histological criteria. Only one of these patients harbored the *CDC73* mutation. The presence of the *CDC73* mutation in about half of the patients with PC reported in the literature, together with its rare occurrence in parathyroid adenomas (7, 8, 14, 16, 26), indicates that it might be involved in PC development and predicts a malignant behavior. Taken together, the current evidence indicates that the finding of a *CDC73* mutation may be an useful diagnostic and prognostic tool, but its absence does not exclude the diagnosis of PC nor a potential malignant behavior. Moreover, the observation that about half of the patients with PC, as well as half of those who had an aggressive tumor, do not carry a *CDC73* mutation raises the question of whether large *CDC73* gene deletions (27) or alterations of its promoter methylation (28, 29), or other yet unknown predisposing genes might be involved.

*CDC73* mutations may impair the expression of parafibromin and its focal/global loss at, as determined by immunohistochemistry, was reported in up to 100% of cases (8, 9, 10, 11, 12, 13, 22, 24). Differences in the rate of parafibromin loss among several studies may be due to the use of different methodologies and scoring systems.

In this study loss of parafibromin was a rather common finding (64%), but the rate of loss was lower than we previously reported (100%) in PC patients who had local invasion and/or metastases at initial surgery or during follow-up (12). Conversely, loss of parafibromin was detected only in one of the ten (10%) patients in the follow-up, whose diagnosis was only based on classic histological features.

Loss of parafibromin was generally associated with *CDC73* mutations, which resulted in a truncated protein. Discrepant results were observed in six cases: loss of parafibromin and no *CDC73* mutation in four tumor samples and the opposite in two. Loss of staining in the absence of mutations detected by direct sequencing of the coding and splice-sites regions could be due to mutations in the promoter, regulatory regions, introns, and 5′ or 3′-UTRs, large whole/partial gene deletions or to abnormalities in the post-transcriptional processing of the protein (27, 28, 30, 31).

As previously discussed for the *CDC73* mutation, the loss of parafibromin in a large proportion of PC indicates that it might contribute to PC development and also predict a malignant behavior. Interestingly, all but one of the eight PC with biologically malignant behavior and retained parafibromin expression did not carry the *CDC73* mutation, suggesting that other genetic abnormalities might be responsible for PC in these cases.

PC has a typically indolent, but progressive, clinical course. Most patients with recurrent disease ultimately succumb to the effects of hypercalcemia, rather than to direct tumor invasion or distant metastases (4). The presence of gross local invasion and/or distant metastases at initial surgery definitely predict a fatal outcome. On the other hand, a complete resection of the primary tumor allows for the greatest likelihood of cure (32, 33, 34). In this study, we confirm that PC has an indolent course in a substantial proportion of patients. As a matter of fact, the 5- and 10-year survival rates were 72.4 and 49.7% respectively. Similar findings were reported by Witteveen *et al*. (10) (60 and 40% at 5- and 10-year follow-up respectively) and Harari *et al*. (32) (78.3 and 66.7% at 5- and 10-year follow-up respectively). In this series, the 5-year survival rate was not predicted by the presence of *CDC73* mutation and/or by the loss of parafibromin in the primary tumor. The rather high survival rate at this time probably accounts for this finding. On the other hand, the loss of parafibromin allowed better prediction of the long-term outcome in individual patients, as the 10-year survival declined to 23% in patients with the loss of parafibromin expression and to 18% when this feature was combined with *CDC73* gene mutation. The parafibromin loss either alone or combined with *CDC73* gene mutation and downregulation of the calcium-sensing receptor expression has recently been reported by Witteveen *et al*. (10) to have a negative effect on the survival rate in a series of 23 patients with PC. At variance with our data, these authors found that the 5-year survival was lower in patients carrying the *CDC73* gene mutation compared with those who did not.

The strengths of our study are that: i) it includes a large series of patients with PC whose histological diagnosis has been established according to the latest WHO guidelines; ii) the median follow-up after PTx was reasonably long; iii) the *CDC73* mutational analysis and parafibromin studies were performed in a single center, thus avoiding potential problems originating from the use of different techniques and immunohistochemical scoring systems. There are also some limitations: i) the *CDC73* mutational screening was confined to the coding and splice sites regions; ii) other putative genes and their protein products could not be investigated because of the limited quantity of available tissue samples.

In conclusion, our data indicate that once the diagnosis of PC is suspected or even established at histology, it would be appropriate to perform parafibromin immunostaining as its loss appears to be an useful tool not only to confirm the diagnosis of PC but also to predict a malignant clinical behavior. *CDC73* mutational analysis does not appear to add value to parafibromin staining in terms of outcome evaluation. Nonetheless, when parafibromin staining is not available, finding the *CDC73* mutation would also predict a negative outcome. Independently of these considerations, the complete evaluation of a patient with PC should include *CDC73* mutational analysis, because the identification of a germline mutation, which occurs in about one-third of patients, would prompt extension of the genetic analysis to other family members.

## Figures and Tables

**Table 1:** Clinical and biochemical baseline data for 35 patients with parathyroid carcinomas^a^.

	
Sex (F/M)	17/18
Age at diagnosis (years)	45±15^b^
Clinical manifestations^c^	
Nephrolithiasis/nephrocalcinosis (*n*=22)^c^	12 (54%)
Osteoporosis/fragility fractures (*n*=22)	17 (77%)
Total serum calcium (mg/dl) (*n*=31)	13.5±2.0^b^
Plasma PTH (pg/ml) (*n*=27)	444 (316, 999)^d^
Follow-up (years) (*n*=35)	7 (4, 11)^d^

a The figure in parenthesis indicates the number of patients with available information.

b Mean±s.d.

c Some patients with clinical manifestations had more than one symptom.

d Median (interquartile range).

**Table 2:** Clinical data and tissue studies of 35 patients with parathyroid carcinomas.

**ID**	**Sex**	**Age at diagnosis** (years)	**Baseline clinical features**			**Tissue studies**^a^				**Outcome**	**Follow-up** (years)
Serum calcium (mg/dl)	Plasma PTH (pg/ml)	Kidney and bone involvement	Cyst features	*CDC73* mutation	Protein annotation	Parafibromin staining (%+ve cells)
(A) Patients with local invasion/recurrence and/or metastases (*n*=25)											
1	F	43	12.4	319	+	No	WT	–	<5	Dead of disease	6
2	F	32	16.0	2000	−	No	c.700C>T exon 7 (G)^b^	R234X	<5	Dead of disease	10
3	M	53	18	1800	+	No	c.195dupT exon 2	N66KfsX16	<5	Dead of disease	5
4	M	50	13.9	384	+	No	c.25C>T exon 1	R9X	<5	Dead of disease	8
5	M	63	12.5	444	+	No	c.197dupA exon 2	N66X	<5	Dead of disease	8
6	F	36	12.0	400	+	No	c.25C>T exon 1	R9X	<5	Dead of disease	6
7	M	45	13.9	350	+	No	c.700C>T exon 7 (G)^b^	R234X	<5	Dead of disease	6
8	M	45	14.0	500	+	No	c.70G>T exon 1	E24X	<5	Dead of disease	16
9	F	52	13.0	1098	+	No	c.415C>T exon 5 (G)^b^	R139X	<5	Dead of disease	2
10	F	56	NA^c^	NA	NA	No	WT	–	<5	Dead of disease	5
11	M	52	16.7	1497	+	No	c.182T>A exon 2	L61X	<5	Dead of disease	2
23	M	60	NA	NA	+	Yes	WT	WT	<5	Dead of disease	2
27	F	56	14.5	554	+	No	c.343G>T exon 4 (G)^b^	E115X	10	Dead of disease	11
							c.64G>T exon 1	G22X			
28	M	40	14.0	96	−	No	WT	WT	<5	Dead of disease	4
34	M	57	NA	2243	NA	No	WT	WT	30	Dead of disease	4
37	M	54	10.8	NA	NA	No	WT	WT	30	Dead of disease	15
38	M	85	13.2	967	NA	No	WT	WT	50	Dead of disease	1
43	M	51	18.8	NA	NA	No	c.60delG exon 1	V20VfsX6	<5	Dead of disease	5
							c.248delT exon 3	I83IfsX26			
21	F	46	NA	NA	NA	No	WT	WT	10	Alive with disease	8
31	M	40	16.0	NA	+	No	NA	NA	<5	Alive with disease	11
32	M	48	11.5	NA	+	No	NA	NA	30	Alive with disease	14
39	F	75	12.2	190	NA	No	WT	WT	80	Alive with disease	2
40	M	71	12.8	722	NA	Yes	WT	WT	30	Alive with disease	4
42	M	54	13.2	1032	+	No	WT	WT	15	Alive with disease	5
12	M	20	15.0	1000	+	No	c.343G>T exon 4 (G)^b^	E115X	<5	Alive free of disease	23
(B) Patients without local invasion/recurrence and/or metastases (*n*=10)^d^											
14	F	76	13.4	410	+	No	WT	WT	10	Alive free of disease	7
16	F	47	10.8	93	+	No	WT	WT	30	Alive free of disease	7
18	F	54	12.6	722	NA	Yes	WT	WT	40	Alive free of disease	1
19	F	68	13.0	312	+	No	WT	WT	30	Alive free of disease	9
22	F	64	12.3	656	NA	No	WT	WT	NA^c^	Alive free of disease	4
24	F	21	13.1	NA	+	Yes	c.343G>T exon 4 (G)^b^	E115X	30	Alive free of disease	10
26	F	73	10.9	96	+	No	WT	WT	10	Alive free of disease	19
29	F	70	12.5	313	+	No	WT	WT	20	Alive free of disease	13
30	M	66	12.2	1300	+	No	NA	NA	<5	Alive free of disease	21
36	F	71	12.7	442	NA	No	WT	WT	10	Alive free of disease	11

a All but one of the studies were performed on the original parathyroid tumors, with the exception being specimen #43 which was a lung metastasis.

b G, germline mutation.

c NA, not available.

d Patients with no evidence of definite local invasion/recurrence and/or metastases during the follow-up.

**Table 3:** Diagnostic value (%) of the presence of *CDC73* mutation and loss of parafibromin immunostaining in the diagnosis of parathyroid carcinoma^a^.

	***CDC73* mutation**^b^	**Loss of parafibromin**^c^
Sensitivity (95% CI)	41 (24–59)	50 (32–68)
Specificity (95% CI)	95 (77–99)	95 (77–92)
Positive predictive value (95% CI)^d^	4 (0–10)	5 (0–12)
Negative predictive value (95% CI)^d^	100 (98–100)	100 (98–100)

a A series of 22 parathyroid adenomas previously characterized for *CDC73* mutations and parafibromin immunostaining was used as a control (Cetani *et al*. (12)).

b CDC73 mutational data were available in 32 patients.

c Parafibromin immunostaining data were available for 34 patients.

d Positive and negative predictive values are calculated given the estimated prevalence of parathyroid carcinoma at our Institution of 0.5% among patients with primary hyperparathyroidism.
